# Understanding Relationships between Health, Ethnicity, Place and the Role of Urban Green Space in Deprived Urban Communities

**DOI:** 10.3390/ijerph13070681

**Published:** 2016-07-05

**Authors:** Jenny Roe, Peter A. Aspinall, Catharine Ward Thompson

**Affiliations:** 1 Center for Design and Health, School of Architecture, University of Virginia, Charlottesville, VA 22902, USA; 2 Stockholm Environment Institute, University of York, Yorkshire YO10 5DD, UK; 3 School of Energy, Geoscience, Infrastructure and Society (EGIS), Heriot Watt University, Edinburgh EH14 4AS, UK; p.a.aspinall@hw.ac.uk; 4 OPENspace Research Centre, University of Edinburgh, Edinburgh EH3 9DF, UK; c.ward-thompson@ed.ac.uk

**Keywords:** ethnicity, poverty, general health, neighbourhood, urban green space, health behaviour, physical activity, social environment

## Abstract

Very little is known about how differences in use and perceptions of urban green space impact on the general health of black and minority ethnic (BME) groups. BME groups in the UK suffer from poorer health and a wide range of environmental inequalities that include poorer access to urban green space and poorer quality of green space provision. This study used a household questionnaire (*n* = 523) to explore the relationship between general health and a range of individual, social and physical environmental predictors in deprived white British and BME groups living in ethnically diverse cities in England. Results from Chi-Squared Automatic Interaction Detection (CHAID) segmentation analyses identified three distinct general health segments in our sample ranging from “very good” health (people of Indian origin), to ”good” health (white British), and ”poor” health (people of African-Caribbean, Bangladeshi, Pakistani origin and other BME groups), labelled ”Mixed BME” in the analyses. Correlated Component Regression analyses explored predictors of general health for each group. Common predictors of general health across all groups were age, disability, and levels of physical activity. However, social and environmental predictors of general health-including use and perceptions of urban green space-varied among the three groups. For white British people, social characteristics of place (i.e., place belonging, levels of neighbourhood trust, loneliness) ranked most highly as predictors of general health, whilst the quality of, access to and the use of urban green space was a significant predictor of general health for the poorest health group only, i.e., in ”Mixed BME”. Results are discussed from the perspective of differences in use and perceptions of urban green space amongst ethnic groups. We conclude that health and recreation policy in the UK needs to give greater attention to the provision of local green space amongst poor BME communities since this can play an important role in helping address the health inequalities experienced by these groups.

## 1. Introduction

Several reviews have recently synthesised evidence showing the public health benefits of contact with green space across a variety of international contexts [1,2] but evidence of benefits by race/ethnicity is a notable gap. Whilst there is a body of literature on inequities of access to urban parks and recreational facilities by race/ethnicity in the USA [3,4,5,6,7], Australia [8] and South Africa [9], the focus has largely been on income inequalities. Evidence of health inequities in relation to access to natural environments in specific ethnic minority groups is very limited. This study aims to fill this gap by exploring how perceptions of urban green space impact on subjective general health across black and minority ethnic (BME) groups in England. In this research the term ”Black and Minority Ethnic” (BME) includes those from Asian, Black or mixed ethnic heritage. This is the most consistently used terminology used in the UK to describe people of non-white descent [10].

Research exploring differences in use and perceptions of urban green space by ethnicity has, to date, been nationally specific, which makes comparisons across different geographies and cultural contexts difficult. Much of the research to date has been carried out in North America, identifying racial/ethnic and socio-economic disparities in urban green space accessibility [11] and differences in use, preferences and motivations in outdoor recreation amongst racially and ethnically diverse groups [12,13]. However, it is difficult to compare such results to European ethnic, cultural and spatial contexts. The pattern of racial segregation in USA neighbourhoods, for example, is on a different scale to that in Europe [14]: city and green infrastructure layout varies by spatial scale, with USA cities more sprawling, having higher levels of car dependency and less street connectivity, which makes comparisons with the European findings on green space and health relationships problematic [15]. For this reason, we have focused our attention on UK and European contexts and ethnicity differences in usage and perception of green space, as the basis for contextualising our study.

### 1.1. Differences in Use and Perceptions of Green Space

Research in England has identified that black and minority ethnic (BME) groups, together with people living in urban deprived areas, choose to access natural environments far less frequently than the average for the white British population [16,17]. This concurs with findings from Commission for Architecture and the Built Environment (CABE) [18] indicating that inequalities in green space provision in BME groups’ residential areas are most likely to account for lower usage patterns, i.e., there is simply less green space available, and it is of poorer quality, compared to other groups’ residential areas. Across a diversity of perspectives, this study showed that white British and BME people from deprived urban communities in England perceived their local neighbourhood open space to be poor in quality (i.e., neglected, offering limited recreational facilities and poor general maintenance). In a further study for CABE [19] the quality of local green space available was found to be strongly related to usage patterns: where people perceived green space quality to be good, and felt safe in it, they were likely to use it more, and were therefore likely to be more physically active, and overall reported better general health.

Understanding what deters BME groups from visiting urban green space, together with concerns about safety, has directed much of the literature to date. Identified barriers include feelings of insecurity due to personal attack or racism; exclusion due to a dominant cultural group; the presence of dogs (i.e., dog fouling or fear of dogs); and poor weather, alongside other demands on recreational time [20,21,22,23]. CABE [19] identified how perceptions of safety differ amongst BME groups, finding, for instance, that only 50% of people of Bangladeshi origin in England felt safe using their local green space, compared to 80% of Indian people, but further in-depth research is needed to fully understand these cultural differences and how they relate to health behaviours such as walking.

There is much less literature identifying what motivates different BME groups to use urban green space and participate in outdoor recreation. The evidence points to different patterns of use and preference by ethnic minorities as compared to mainstream white populations but has failed to identify the underlying–and intrinsic–motivations behind these behaviours. In England, Rishbeth [24] found some BME groups (i.e., Asian British/African-Caribbean) were less likely to use a park for exercise when compared to white British. CABE [19] found the opposite pattern, with some BME groups more likely to visit for physical activity (i.e., people of Bangladeshi, Indian and Pakistani origin), whilst white British were more likely to visit for rest and relaxation. There is therefore a need to understand motivations for day-to-day outdoor health behaviours from different cultural perspectives.

One consistent finding across the literature is that non-Western immigrants are more likely than the native European population to use urban green space for social gatherings [25]. Visits mostly take place with someone else, as opposed to visiting alone [4] and popular activities relate to food, such as picnics, barbecues and fruit picking [19,20,24,25] and in large family groups, most often in the evenings [23]. There appear to be gender differences in social behaviours, with women from varied ethnic groups—including first generation immigrants—more likely to visit urban green space with family and friends [19,25,26,27]. It is posited that ethnic groups place more value on their families [25] but this theme needs to be much better understood in relation to health outcomes and behaviours. Precisely why ethnic minorities visit urban green space is not well understood nor is any relationship to health and wellbeing.

### 1.2. Ethnicity Trends in the UK

The 2011 decennial National Census highlighted increased ethnic diversity in England and Wales, with a 7% drop in the majority white population and increases in all ethnic groups, with the biggest rise of 2.5% representing an increase in the Asian/Asian British population (e.g., people of Pakistani, Indian, Bangladeshi origin). The greatest transition in ethnicity is within cities, with increased BME populations of up to 13% in London, Manchester and Birmingham [28]. At the same time, BME people in England suffer from greater income, health and environmental inequalities, on average, than white British people. Many BME people are living in England’s most deprived census wards (as measured by the Indices of Deprivation), populated with—on average—four times more BME people than more affluent wards [18]. Persistent inequalities are seen in the general health of BME groups, with the percentage of the population reporting poor general health highest amongst people of African-Caribbean origin (23% reporting “not good” health), followed by Bangladeshi and Pakistani ethnic groups (18% in “not good” health). Ethnic inequalities in health are more pronounced by gender and older age, with persistent inequalities seen in women of Pakistani and Bangladeshi origin, and 56% of all women from ethnic minorities aged 65 or older reporting a limiting long-term illness [29]. These health disparities stem from economic determinants, education, geography and neighbourhood environment (e.g., access to healthy food outlets and recreational facilities, air and noise pollution), reduced access to health care services and the chronic stress that arises from poverty and discrimination [30]. It has also been suggested that inequitable health outcomes result from inequitable distribution of, or access to, natural environments that promote good health outcomes [31] but exploration of this in racial and ethnic minorities is limited.

### 1.3. Background to the Study

Using a self-rated general measure of health [32], this study explores individual, social and environmental predictors of general health in six ethnic groups living in the three of England’s most ethnically mixed conurbations (i.e., London, Manchester and the Wolverhampton and Coventry area) with a particular interest in understanding the dynamics of place (i.e., social perceptions and physical attributes of place) and their relationship to general health. It builds on earlier research commissioned by CABE [18,19] carried out by the authors in conjunction with Heriot Watt University, which highlighted inequalities in access to green space amongst those living in deprived inner-city areas in England. This study underlined that, until now, very little has been published about the impact of these environmental inequalities on the general health and wellbeing of specific BME groups.

Given the substantive evidence showing public health benefits of urban green space in deprived urban environments [33,34]—and the poorer general health of BME groups [32]—it follows that BME groups would likely benefit from engaging with urban green space. However, the earlier CABE study reported that the most deprived 10% of census wards (the unit of analysis defined by electoral boundaries) showed on average only a fifth of the area of green space per ward that is available to the most affluent 20% of wards [18]. This study also showed that in areas most densely populated by black and minority ethnic groups (i.e., comprising 40% plus of population in a ward), there is not only less green space, but it is of poorer quality (i.e., neglected, offering limited recreational facilities and suffering from poor general maintenance).

In summary, very little research has explored how perceptions of urban green space by BME groups can be explained by demographics such as gender, age or disability, and is inconsistent in its findings in relation to motivations for health behaviours such as physical activity. Klock et al. [21], synthesizing research on BME usage and perceptions of green space in Northwest Europe (i.e., the UK, The Netherlands and Germany), conclude that the research has failed to properly explore explanatory factors for these perspectives, particularly in relation to gender and age. Empirical evidence of inequalities in green space access for BME groups in England is limited to only a handful of studies [17,18]. Whilst urban green space behaviours appear to vary by ethnicity and context, the reasons for these behaviours and their relationship to health is not understood. No research has yet linked access to neighbourhood green space with health outcomes amongst BME groups in England. This study aims to fill this gap in a sample of white British and BME groups living in deprived urban locations in England.

### 1.4. Research Questions

For people of varying ethnicity living in deprived urban neighbourhoods in England:

RQ1: how does general health differ between ethnic groups and what are the distinguishing health profiles?

RQ2: how does use and perceptions of neighbourhood environment/urban green space differ between ethnic groups?

RQ3: what demographic, social and physical attributes of place predict general health amongst different ethnicity general health profiles?

## 2. Materials and Methods

### 2.1. Choice of Sample Locations

The sampling structure was developed to reflect the geographic concentrations of particular BME groups in England, with a focus on metropolitan areas in the Midlands, Greater Manchester and London. Firstly, case study locations were selected using one or more 2001 census wards as the area or locational unit in which sampling would take place. Locations were chosen using the following criteria:

- Wards classed among the most deprived 20% in England (Index of Multiple Deprivation (IMD) of 1 or 2) [35].
- Wards with a minimum of 9% of the population from black and minority ethnic (BME) groups (according to the 2001 Census) [28].
- Wards with not less than 20% and not more than 45% land use classified as areas of green space (derived from 2005 Generalised Land Use Database (GLUD) [36].
- Wards with varying quality indicators for green space (e.g., Local Authority (LA) performance in relation to green space) (2005 GLUD) [36].

This resulted in the selection of 6 case study locations in London (Hackney and Islington), West Midlands (Coventry and Wolverhampton) and Greater Manchester (Rochdale and Oldham).

### 2.2. Sampling Strategy

Using a quota sampling method to capture specific sub-groups, a sample of 80–85 adult participants (aged 16 and over) was obtained from each location, to include at least 40 participants from each of six ethnic group categories: people of African-Caribbean, Bangladeshi, Indian, Pakistani origin, Other BME and white British (see Appendix A for full sampling structure). The survey was undertaken in May 2009 and was administered by a market survey company, Ethnic Focus. It was carried out as a computer-assisted interview, face-to-face, with one person in each household chosen, and was approximately 45 min in duration. Participants were offered a small token of thanks for their contribution in the form of a voucher.

Ethnicity was self-reported using the 2001 census classifications (i.e., 14 ethnic group classifications in total). Given we had targeted six specific ethnicities, and having reached the target quota (see above), participants’ ethnicity was classified into six ethnic groups. For example, African-Caribbean (8% of our sample) and black African (5% of our sample) participants were combined into one group (*n* = 63) (hereafter referred to as people of African-Caribbean origin). ”Other” BME groups included a diverse range of people (e.g., people of Chinese origin (2% of sample) and Other White Background (2% of sample)). In each case, this was a pragmatic solution owing to the comparatively small sample numbers in each sub-group. Location was generally a proxy for a concentration of white British or BME in our sample: white British (Islington, Coventry and Wolverhampton), African-Caribbean (Hackney), Bangladeshi (Oldham and Rochdale), Pakistani (Oldham and Rochdale), Indian (Coventry and Wolverhampton) and “other” ethnic groups (Hackney and Islington).

### 2.3. Measures

#### 2.3.1. Individual Measures

- Demographic measures: In addition to age, gender, ethnicity, and religion, self-report data were collected on socio-economic indicators that included indicators of income coping, type of housing tenure, educational attainment, current work status, number of children, car access and disability.
- Self-Reported Health Measures:
  - General Health: the primary outcome measure in our study, was a single-item scale asking participants to rate their general health, ranked on a 5-category Likert scale from 1 (very good health) to 5 (very poor health). This is a valid and reliable measure of subjective health [37].
  - Physical activity level: measured using one item asking for the number of days on which physical activity (of sufficient exertion to raise breathing rate) reached or exceeded 30 min, recalled over the past 4 weeks, based on 2008 recommendations from the British Heart Foundation National Centre [38].

#### 2.3.2. Social Environment

c. Perceptions of Loneliness: a 3-item loneliness scale [39] comprising measures of companionship, feeling left out, and isolation, each rated on a 3-category scale from 1 (low level of loneliness etc.) to 3 (high level of loneliness etc.);
d. Perceptions of Place belonging: one item scale [40] rated using a 5-category scale (i.e., from 1 ”strongly disagree” to 5 ”strongly agree” on belonging);
e. Perceptions of Neighbourhood Trust: two items on trust [40], one item measuring general levels of trust in the wider neighbourhood, the other levels of trust in leaving a key with a neighbour, each ranked on a 3-category scale (1 = high levels of trust to 3 = low levels of trust).

#### 2.3.3. Neighbourhood Environment

f. Perceptions of the overall neighbourhood: measured using two items, one item on general Satisfaction with the neighbourhood as a place to live (ranked on a 5-item scale from high satisfaction (1) to poor (5)); the other item on the likelihood of recommending the area to a friend as a place to live (ranked from ”yes definitely” (1) to ”no, definitely not” (5)), which we have labelled Liveability of the neighbourhood.

#### 2.3.4. Local Green Space

g. Perceptions of local green space quality: measured using three items (i.e., safety, attractiveness, satisfaction with urban green space), with quality on all questions ranked on a 5-item Likert scale from high (1) to poor (5).
h. Self-reported use of local green space: current usage of nearest neighbourhood green space was measured using self-reported frequency of visits over summer and winter. We also asked about the social dimension of these visits, i.e., ”with whom” visits were made (alone or with friends/family). In addition we asked about how the nearest neighbourhood green space was accessed (i.e., walking, car, public transport) and the availability of a 2nd local urban green space.

### 2.4. Approach to Statistical Analysis

Firstly, using SPSS 18, we ran descriptive statistics to identify characteristics in the population, to explore differences in general health between white British and BME groups, and to identify different usage patterns of neighbourhood green space (RQs 1 and 3). We used non-parametric tests such as Mann Whitney U and Kruskal Wallis depending on the number of comparisons.

Secondly, we used Chi-Squared Automatic Interaction Detection (CHAID) segmentation to provide sub-group profiles within the sample based on the health variable, general health (RQ1). This method of analysis was selected based on the following rationale: conventional regression analysis implicitly assumes that the predictors from a sample can be generalized to the population; however in situations where the sample contains sub-populations this assumption is unrealistic and conventional regression may produce biased estimates [41]. In such circumstances the use of regression mixture (or latent class) models provides greater flexibility in allowing regression parameters to vary within each sub-class within the data. You had two different references as 41 in original reference—might be worth checking that these are right (not sure if some are referring to the same reference/which one is which) now 42 and 43 in references.

The CHAID algorithm provides one such approach by profiling latent segments (i.e., subtype profiles) in a tree structure which can include classification for nominal or ordinal dependent variables [42]. CHAID also allows for more than two branchings at any point in the tree at which there are significant differences. When compared with other inferential statistical methods, the CHAID algorithm has additional advantages. First, as Green and Salkind [43] point out, CHAID does not require the assumptions for running typical inferential statistical analysis. Second, while inferential statistics evaluate only whether there exists a significant difference among mean scores of dependent variables in each category of independent variables, the CHAID algorithm makes decisions about dependent variables at each terminal node (i.e., the endpoints) as the tree progresses. In the approach taken here, a CHAID analysis was initially applied to the dependent health variable ”general health”.

Having established the main health segments, this was followed by a new form of high dimensional Correlated Component Regression (CCR) with M-Fold Cross Validation. This is one of a number of methods which have been developed to regularise regression for linear modelling as a means of reducing prediction error. In this case regularization is achieved through component/dimension reduction strategies as shown by Magidson [44]. The purpose is to optimise *R* squared by manipulating both the number of correlated components (*k*) and the predictors (*p*) in any model. Whereas in conventional regression, *k* is fixed at *k* = *p*, Magidson shows that this saturated model fit is frequently improved when *k* < *p* leading to more parsimonious models with less variance in estimates over different samples. Another unique feature of CCR is that *p* values are replaced by cross validated out-of-sample performance as provided in the predictor tables.

In determining the final number of predictors a standard error rule was applied. This means that either the optimum number of predictors (as determined by the maximum value of *R* squared) was selected or in situations where smaller numbers of predictors fell within one standard error of the optimum then the predictor number closest to one standard error from the optimum was selected i.e., a parsimonious solution [44].

To assess the relative importance of each of the predictors to the dependent variable, the Pratt measure was used as this compensates for collinearity between correlated predictors in beta weight estimations [45]. In Pratt’s importance measure, the unique contribution of any predictor to the final regression model is obtained by multiplying its beta weight by its zero-order correlation with the dependent variable (i.e., its correlation with the dependent variable in isolation from other predictors).

## 3. Results

RQ1 what individual characteristics distinguish between ethnic groups and how does general health differ between them?

Between-group differences (Kruskal Wallis and Mann-Whitney means tests) are reported in Table 1 below. With the exception of age, our ethnicity groups significantly varied on all demographic and SES variables.

General health and levels of daily physical activity also significantly varied between groups: people of Bangladeshi, Pakistani, African-Caribbean origin and Other BME groups had significantly lower levels of general health (as compared to the average in our sample); people of Bangladeshi and African-Caribbean origin also had significantly lower levels of physical activity than average, with especially low levels in people of African-Carribbean origin (3.6 days/month compared to mean of 7.4 days/month for the sample).

**Figure 1:**
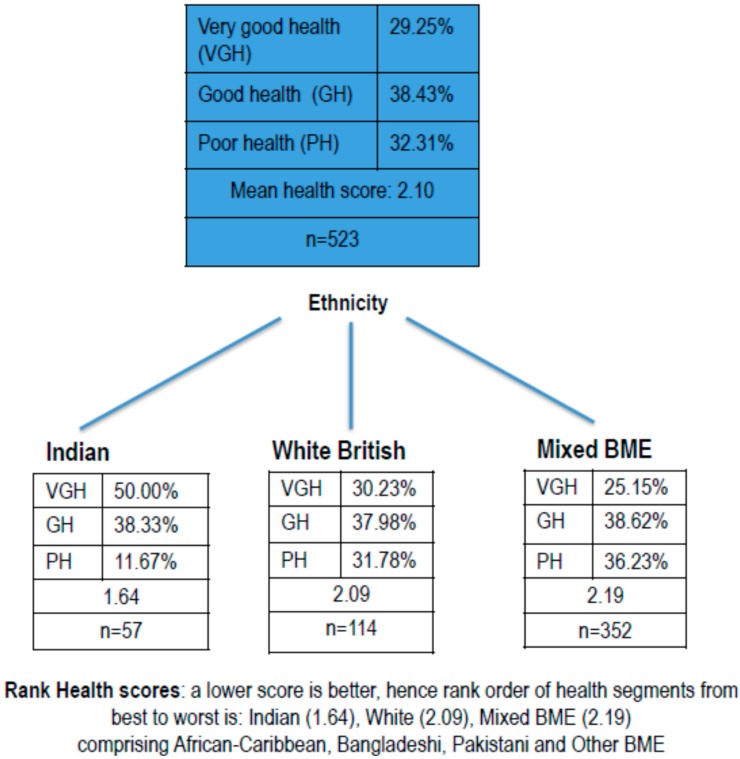
Chi-Squared Automatic Interaction Detection (CHAID) segmentation of data by general health.

To explore between-group differences in health, we next ran CHAID segmentation analyses using general health (GH) as the dependent variable. As reported above, we used the Decision Tree algorithm (SPSS 18). The first split in the tree node indicates ethnicity as a predictor of general health (GH). The analyses identified three distinct health segments: people of Indian origin, white British, and a single segment combining people of African-Caribbean, Bangladeshi, Pakistani origin and Other BME (see Figure 1 below). This last segment has been labelled “Mixed BME” in the subsequent analysis described below, for ease of reference. In Figure 1, the rank order of health segments (i.e., health score), reading left to right at the lower level, from best to worst health, is Indian (mean GH 1.64), white British (mean GH 2.09), and Mixed BME (mean GH 2.19) (note a lower score indicates better general health).

The Chi-square test of independence indicated there was a significant difference in health between the 3 segments of ethnicity which have been identified in the tree structure (Chi-square = 21.11, df = 2, *p* < 0.001). This was the segmentation profile used to explore RQ3 and to identify individual, social and physical environmental predictors of general health.

RQ2: how do ethnic groups differ in their perceptions of place characteristics? (Table 2)

Between-group differences (Kruskal Wallis) in perceptions of neighbourhood environmental attributes are reported in Table 2 below. Results show that ethnic minorities’ perceptions of the neighbourhood, together with their use and perception of urban green space, significantly differ on all of our place attributes. Explored by health segmentation, the ”best” health group, people of Indian origin, rate their neighbourhood more positively than Mixed BME (but of similar rank to white British) and feel greater levels of place belonging, trust and less loneliness than either white British or Mixed BME groups. People of Indian origin are most likely to visit their local urban green space with someone (89.9%) and by walking (81.5%). By comparison, BME in the ”worst” health group (i.e., people of African-Caribbean, Bangladeshi, Pakistani origin and Other BME)—have much more negative perceptions of local place characteristics. They are less satisfied with the neighbourhood environment, less satisfied with urban green space quality (i.e., safety and attractiveness), and much less likely to visit in winter and summer. This is particularly pronounced in people of Bangladeshi origin.

RQ3: what individual and place characteristics predict general health amongst different ethnicity health profiles?

We ran three CCRs to establish a set of predictors for each general health segment (people of Indian origin, white British and Mixed BME) as set out in Table 3 below. Full detail on the regressions is provided in the Appendix B (see Table B1, Table B2 and Table B3), including the relative importance of each variable in making a % contribution to the model. The table illustrates the different groupings of predictors for each health group with the rank order in terms of predictor power.

A full list of predictors entered into the CCR analysis is as follows: Demographics (gender, age, marital status, disability, no. of children); Socio-economic status (SES): education, current work status, income coping, housing tenure, car access; Physical activity; Social environment (neighbourhood trust, place belonging, loneliness); Neighbourhood environment (satisfaction with the area and liveability); and Local green space (green space quality, use of green space).

Individual predictors of general health: across all groups (best to worst health profiles), consistent predictors of general health—with highest rank order—were physical activity and age. Being physically active and younger in age were associated with better health across all health profiles. Being in work was associated with better health in people of Indian origin and Mixed BME profiles. Having no disability was associated with better health in white British and Mixed BME profiles. Being female was associated with better health in only the poor health (i.e., Mixed BME) profile. With the exception of work status, other SES variables (i.e., subjective income coping, education, tenure) were not significant predictors of health in the models.

Social environment: across all groups, perceptions of the social environment predicted general health, but the dynamics varied by health segment. Trust in neighbours was a highly ranked predictor of health in people of Indian origin (best health group). Place belonging was a predictor of general health in white British (good health) and Mixed BME (poor health) only. An interesting finding is that the high predictor ranking of the three loneliness variables (i.e., companionship, feeling left out, isolation) was amongst the white British segment (good health) only. Loneliness amongst this segment is a significant discriminator of general health, but the pattern is not present in other BME health groups (i.e., Indian and Mixed BME).

Neighbourhood environment: a consistent predictor of general health across all groups, and highly ranked, is neighbourhood satisfaction as indicated by one of two variables entered in the model (i.e., satisfaction levels, liveability of the area).

Local green space: satisfaction with local green space and perceptions of safety were associated with better health in the Mixed BME group only. Perceived green space attractiveness was not a significant predictor.

Secondly, easy access to green space (e.g., by walking) was associated with better health in people of Indian origin (very good health) and white British (good health). Visiting a local green space with someone was associated with better health in the Mixed BME group only (poor health). Visiting local green space more frequently in winter was also associated with better health in this last group, but this was not true for summer visits. Perceived distance to green space from home was not a significant predictor.

In summary, results show individual and place characteristics consistently discriminate between general health levels across all three ethnicity health segments. Perceived quality of the neighbourhood is a consistent predictor of general health across all groups; the quality of green space emerged as a significant predictor in the worst health group i.e., Mixed BME. Visiting patterns to local green space (i.e., means of access, frequency of use and social patterns of use) vary between groups, with the social dynamic of visits emerging as a notable predictor of general health in Mixed BME.

## 4. Discussion

This study explored differences in general health amongst white British and BME groups and how individual and place context influence these differences. The findings reported here build on a wider investigation of related issues, commissioned by the Commission for Architecture and the Built Environment [18,19].

Firstly, we found levels of general health were highly variable amongst our ethnic groups, with three general health profiles emerging from “best” to “worst” health. Whilst we made no hypotheses in relation to these groupings by BME, it is not surprising, given the literature on health inequalities, that the “good health” group comprised white British people and the “worst health” group the majority of our BME groups (notably people of African-Caribbean, Bangladeshi and Pakistani origin). This is consistent with UK statistics on self-reported general health, which show poorer general health in all three of the above BME communities [28,32]. What is surprising, given UK health population statistics, is that people of Indian origin reported “best” general health, since, on average, people of Indian origin tend to report similar general health to white British [28,32].

The above finding was matched by highly variable levels of physical activity. Overall, we found very low levels of physical activity among our white British and BME groups, reflecting trends recently reported by Koshoedo et al. [46] but this significantly varied by health group. For example, people of Indian origin in “best health” reported exercising on average 9 days per month and, whilst not high for the average general UK population, this was significantly higher than the rest of our sample. We found markedly different patterns of physical activity in winter amongst people of Indian origin and Mixed BME groups, with frequency of visits to green space in winter statistically lower than in summer. This suggests that health promotion directed at encouraging BME to be more active outdoors in winter may positively impact on health. However, there is a need to better understand patterns of physical activity in light of cultural differences, health barriers, climatic barriers and gender restrictions on mobility outdoors [46,47].

Secondly, we found perceptions of place context and of the “liveability” of the neighbourhood significantly varied between white British and BME groups. This was consistent with the literature, showing, for example, lower levels of perceived neighbourhood satisfaction, together with lower levels of place belonging and trust among BME communities, particularly people of African-Caribbean, Bangladeshi and Pakistani origin [19]. Those people of Indian origin in our sample again countered trends in previous evidence for people of Asian British origin, reporting high levels of satisfaction with the neighbourhood environment, greater perceived quality in urban green space (i.e., safety and satisfaction) and higher usage levels (in summer only) than our other groups.

Thirdly, predictors of general health varied by ethnic group.

### 4.1. Individual Characteristics

The most consistent predictors of general health across all three health groups were physical activity and age; given that the greatest variation of general health by ethnicity is seen among older people [32] this is not surprising. We found interesting sub-group patterns by age. For example, people of African-Caribbean origin aged 65+ reported the poorest health of any group in our sample (mean = 4.5) compared to younger people of the same ethnic origin, aged 16 to 24, who reported good general health (mean = 1.72). Greater understanding of the determinants of health across the life span and generations in specific BME groups is therefore needed.

It is not surprising that disability was a predictor of poorer health, nor that gender was a determinant of “worst” health in Mixed BME, given the poorer reported general health of women of Pakistani and Bangladeshi origin elsewhere [32]. Of our individual variables, neither subjective income coping nor living circumstances (e.g., housing tenure, deprivation indicators) were significant predictors of general health. This is surprising given the evidence of relationships between health and SES variables such as housing tenure, with private renters typically having poorer health [48,49] and financial hardship consistently predicting self-reported health [50]. Of our SES variables, only work status was a predictor of general health, and only in BME groups. Our Indian profile—in “best” health—were more likely to be in paid work than other BME groups, whereas people of Bangladeshi and Pakistani origin were much more likely to be not in work for some reason. The results indicate underlying complexities in SES factors, ethnicity and health relationships.

### 4.2. Place Characteristics: Social

Across all three health segments, perceptions of the social environment (i.e., place belonging, level of trust in neighbourhood, loneliness (i.e., companionship, feeling left out, isolation) determined general health in one or more combination of variables, but the concentration of these predictors was highest among white British people, where feelings of trust and place belonging were below the average for our sample. Perceptions of the social environment appear to be significantly more important to the health of deprived urban white British people than other BME groups in our sample. This might reflect the fact that deprived urban neighbourhoods in the UK are frequently in a state of transition, particularly in London (where the majority of our sample of white British originated and where ethnicity flux is greater than anywhere else in the UK). Poorer people, especially in private rented homes, suffer from displacement and a lack of security in tenure, a dimension that is known to impact negatively on wellbeing and prevents firm social bonds from developing [51]. It is also possible, given the reported collectivist values of British Asian families [52]—and the strong family ties and social support—that the home, rather than the wider neighbourhood, is fostering belonging and social cohesion in these ethnic groups to a greater degree and, in turn, improving their health outcomes.

### 4.3. Place Characteristics: Environment

Across all health groups, a consistent and highly ranked predictor of general health is the “liveability” of the area, as ranked by satisfaction levels with the area, and the likelihood of recommending a friend to live there. This is consistent with other research in deprived urban communities showing strong relationships between neighbourhood satisfaction and quality of life [19,53]. Higher quality perceptions of the “liveability” of a neighbourhood was a predictor of better general health in people of Indian origin and white British groups. Our findings extend those of Karlsen et al. [54] which showed an effect of perceived place quality on self-reported health in British ethnic minorities (i.e., people of African-Caribbean, Indian, Pakistani and Bangladeshi origin) but was unable to identify what precise indicators of neighbourhood quality have an effect. By including a wider range of neighbourhood quality indicators in this study, including measures of urban green space, our study gets closer to identifying what area level indicators are important in ethnicity, environment and health relationships.

Significant predictors of self-reported health included urban green space quality indicators (safety, attractiveness, satisfaction) alongside usage patterns, but the characteristics varied by ethnic group. Walking to local urban green space was a predictor of the “best” health group—people of Indian origin—who also reported higher safety perceptions, higher quality green space, and were much more likely to be accompanied by someone (90%), but these latter variables were not significant predictors of general health. Similarly, “good” health in white British people was predicted by walking to local urban green space and the presence of a second neighbourhood green space, also accessed by walking. This reflects findings from our earlier study showing the importance of local green space in deprived urban communities [19].

General health in our “worst” health group (i.e., ethnicities within the Mixed BME group) was far more likely to be predicted by urban green space usage and perception variables than in any other ethnic group. This is a key finding, suggesting that health policy needs to better understand how to use urban green space in fostering positive health behaviours in the these ethnic groups throughout the year. Communities within the Mixed BME group perceived the quality of local green space to be poorer (i.e., less safe, less attractive) and their visiting patterns were significantly different between winter and summer, with a low level of visits in winter a significant determinant of poor general health. The trends are particularly pronounced for people of Bangladeshi origin.

An interesting finding is that the social dynamic of visiting urban green space (i.e., with someone rather than alone) varies in its relationship to general health in BME groups. It was a predictor of health only in our “worst” health group—Mixed BME—and not among people of Indian or white British origin. Based on the strong evidence of social visiting in BME groups [24,25,26] we might have predicted this variable would consistently determine general health across all BME groups. That we found this variable significant in our “worst” health group only indicates the complexity of the social determinants of general health—and their relationship to the environment—amongst ethnic groups and across cultures. For instance, we know young Asian British women will only be likely to visit local green space if they can find a space to be among women of their own ethnicity [19]. There is therefore a need to understand these relationships at a local level and for UK park providers and recreational facilitators to understand these patterns of behaviour much better to steer provision for BME groups more appropriately.

In summary, our study found that general health—and individual, social and environmental predictors of general health—significantly differ among ethnic groups. This suggests that improving the general health of economically deprived, ethnically diverse urban communities needs to consider the local context very carefully and sensitively prior to implementing any neighbourhood change and/or recreational facilitation designed to improve health. For instance, different BME communities have quite distinct perceptions and patterns of use in urban green space, and their motivational reasons for use vary. There is therefore a need for planners and policy makers to engage with BME groups at a local level, to explore their specific needs and appropriately target local facility provision. In addition, further qualitative and quantitative research is needed to develop a better understanding of use and attitudes within specific BME groups, particularly in relation to gender and age. Owing to the continuing dynamic nature of ethnicity in the UK, engagement with these communities is likely to be required on an on-going basis, since patterns of behaviour and use are likely to change over time, both between generations and owing to current shifts in migration patterns and age demographics.

In a wider geographic context, our findings contribute towards an understanding of the need for a culture of health specific to locality and race and ethnicity. A “culture of health” is broadly defined as a culture that supports health improvement by fostering healthy, equitable communities that enable everyone to make healthy lifestyle choices [55]. The growing diversity of our cities, from both planned and unplanned migration, is transforming how health is understood and negotiated [56]. Integral to this is the need to understand how perceptions of health and wellbeing differ across racial and ethnic minority groups, and how those differences impact on people’s ability to foster positive health behaviours at work, home and within the community. The current study has shown how perceived place characteristics—both social and physical environmental attributes—vary by culture, and how this impacts general health.

### 4.4. Limitations

Owing to small numbers of “other BME” (e.g., Chinese, white European) we had to aggregate some of our data, therefore losing distinctiveness across some ethnic groups, and forcing generalisations to be made in this group. We were also limited in our “best” health group (people of Indian origin (*n* = 57)) to explore differences by gender and age. We would recommend larger sampling in future, in order to explore intra-ethnic differences in more depth.

Synthesis of the findings from our study with those from other countries is difficult to achieve owing to differences in scale, context, culture and geography. However, as Klock et al. [21] recommend, crossing borders can illuminate common problems in the research and help establish conceptual frameworks for cross-cultural studies. Migration is an international phenomenon and international research, for example, on the role of urban green space in fostering social inclusion, is particularly important research to accelerate, especially in the current context of forced migration within Europe. It is in research on immigrant populations where cross referencing between such studies might be best be focused. For example, in North America, research has suggested that activities in the natural environment serve as a protective factor in the health and well-being of immigrant families, providing emotional and physical health benefits that can help buffer stressors such as inadequate housing, emotional stress and displacement [57]. This warrants further exploration in European contexts.

All the data (with the exception of demographics) are self-reported, and therefore subjective in nature. Whilst our dependent variable, self-reported general health, reflects metrics used in UK Census statistics, it would be helpful to explore how general health interacts with wellbeing (e.g., perceived stress) and objective biomarkers of health (e.g., heart rate, cortisol) in the future. Future research should also aim to gather objective data on access to and quantity of available green space, with which to correlate the health data, to provide a further basis for interpretation of findings and their implications for policy.

Drawing on the models for a ”culture of health” we would also suggest that the meaning of health and wellbeing is explored with specific BME groups and appropriate metrics generated from this research specific to local context and culture. Whilst we explored health and wellbeing in the qualitative work that accompanied this research [19] we used a standardized metric of general health to compare findings with the wider population. We recognize, however, that health is tied to place and we need new models of health and metrics to capture what it means for specific cultures.

As far as we know CCR is the only regularisation model available for non-linear models (e.g., logistic regression). A limitation therefore is that at this stage more work is needed in comparing CCR with other cross validated approaches. However on the basis of the published work by Magidson [44] CCR performs extremely well, particularly in small samples and also in highly collinear problems which is frequently the case in questionnaire data as here.

## 5. Conclusions

This study adds to the existing evidence base indicating the role of place attributes—including urban green space—in determining general health in deprived urban communities, and, for the first time, extends these findings to black and minority ethnic groups in England. However, how these place attributes impact on general health varies according to ethnicity, with those BME groups in worst health more susceptible to the quality of urban green space. The details also appear to vary according to individual circumstances such as gender, age and work status. Whilst further research is required to confirm these trends, it suggests that there is an opportunity to support better health in some of the most deprived urban populations in England by enhancing the environment, and green space in particular, and encouraging its appropriate use by BME groups. Future provision—and facilitation in use—of urban green space in deprived urban communities explores cultural differences within a local context and is recommended to identify how health-improving behaviours can best be supported.

## Figures and Tables

**Table 1:** Individual characteristics of six ethnic groups, *n* = 523.

Characteristics			Total *n* = 523	White British *n* = 114	Indian *n* = 57	African-Caribbean *n* = 63	Bangladeshi *n* = 89	Pakistani *n* = 115	Other BME *n* = 85	Between Group Statistical Difference
Percentage sample			100%	23%	11%	12%	17%	23%	16%	
**Demographics measures**	Age (years) n%	≤44	70%	66%	64%	75%	70%	70%	83%	ns
		≥45	30%	34%	36%	25%	30%	30%	17%	
	Gender (m = male, f = female) n%		M = 40% F = 60%	M = 53% F = 47%	M = 31% F = 69%	M = 44% F = 56%	M = 30% F = 70%	M = 38% F = 62%	M = 38% F = 62%	*
	Income coping ^a^ M (SD)		2.66 (1.24)	2.44 (1.19)	2.36 (1.20)	2.68 (1.35)	2.76 (1.25)	3.01 (1.15)	2.60 (1.25)	**
	Current work status: n% not in work for any reason		51%	50%	34%	40%	68%	51%	40%	**
	Disability: n%		5.2%	7%	1.7%	7.9%	6.7%	4.5%	1.4%	ns
**Health measures**	General Health ^b^ M (SD)		2.10 (0.92)	2.08 (0.93)	1.63 (0.69)	2.22 (0.99)	2.27 (0.86)	2.26 (0.98)	2.23 (0.84)	***
	Physical Activity M (SD) (days/month)		7.4 (7.4)	8.2 (6.8)	9.0 (7.83)	3.6 (3.9)	7.0 (7.32)	7.8 (8.5)	7.7 (8.08)	***

Notes: Abbreviations: Mean (M), Standard Deviation (SD), ns = non-significant; **^a^** a lower score indicates higher perceived income coping; **^b^** a lower score indicates better general health; *** *p* < 0.001, ** *p* < 0.01, * *p* < 0.05; n% is quoted for within ethnicity group sample, columns 3 to 8.

**Table 2:** Perceptions of place by six ethnic groups.

Environmental Indicators		Total *n* = 523 M (SD)	White British *n* = 114 M (SD)	Indian *n* = 57 M (SD)	African-Caribbean *n* = 63 M (SD)	Bangladeshi *n* = 89 M (SD)	Pakistani *n* = 115 M (SD)	Other BME *n* = 85 M (SD)	Between Group Statistical Difference
**Social Environment**	Loneliness ^a^ M (SD)	1.37 (0.49)	1.35 (0.46)	1.26 (0.42)	1.51 (0.53)	1.45 (0.51)	1.32 (0.47)	1.40 (0.53)	***
	Place belonging ^a^ M (SD)	2.04 (0.94)	1.77 (0.82)	1.58 (0.75)	2.18 (1.04)	2.46 (0.87)	2.09 (0.91)	2.25 (1.06)	***
	Neighbourhood Trust ^a^ M (SD)	2.30 (0.88)	2.22 (0.73)	1.86 (0.68)	2.41 (1.19)	2.49 (0.66)	2.40 (0.75)	2.30 (1.08)	***
**Neighbourhood Environment**	Satisfaction with the area ^a^ M (SD)	2.02 (0.92)	1.77 (0.07)	1.76 (0.12)	2.38 (0.13)	2.18 (0.09)	1.98 (0.81)	2.26 (0.13)	***
	Liveability ^a^ M (SD)	2.00 (0.99)	1.69 (0.7)	1.75 (0.15)	2.11 (0.13)	2.24 (0.09)	2.21 (0.09)	2.10 (0.13)	***
**Local Green Space (GS)**	GS Satisfaction ^a^ M (SD)	2.10 (0.88)	1.82 (0.06)	1.83 (0.09)	2.11 (0.10)	2.61 (0.11)	2.24 (0.96)	1.99 (0.09)	***
	GS attractiveness ^a^ M (SD)	2.06 (0.85)	1.74 (0.64)	1.81 (1.60)	2.11 (0.68)	2.49 (0.99)	2.2 (1.02)	1.99 (0.72)	***
	GS Safety ^a^ M (SD)	2.25 (0.88)	2.13 (0.07)	2.14 (0.09)	2.03 (0.10)	2.55 (0.10)	2.39 (0.87)	2.13 (0.12)	**
	GS Access: n% walking	72.3%	75.1%	81.5%	75.1%	70.8%	80.5%	85.9%	ns
	GS Social use: n% visiting with someone	74.8%	81.4%	89.9%	73.02%	71.91%	83.2%	84.1%	*
	GS availability: n% with access to a 2nd local GS	43.2%	55.58%	55.9%	28.6%	31.5%	39.1%	43.4%	***
	GS frequency visits (winter) ^b^ M (SD)	5.27 (1.63)	4.71 (0.14)	5.12 (0.19)	5.35 (0.20)	6.19 (0.15)	5.42 (0.15)	4.97 (0.20)	***
	GS frequency visits (summer) ^b^ M (SD)	3.83 (1.74)	3.07 (0.12)	3.29 (0.19)	3.62 (0.20)	5.18 (0.19)	4.15 (0.18)	3.68 (0.20)	***

Notes: Abbreviations: Mean (M), Standard Deviation (SD), ns = non-significant, GS = Green Space; **^a^** a lower value indicates higher perceived quality on neighbourhood and local green space variable and higher ranked perceptions of the social environment (i.e., less loneliness, greater place belonging and greater trust in neighbourhoood); **^b^** a higher value indicates fewer visits to GS; *** *p* < 0.001, ** *p* < 0.01, * *p* < 0.05; n% is quoted for within ethnicity group sample, columns 3 to 8.

**Table 3:** Predictors of general health by health group (rank order of predictors shown in parenthesis).

Predictors	Group 1 Indian Very Good Health (*n* = 57) 7 Predictors	Group 2 White British Good Health (*n* = 114) 11 Predictors	Group 3 Mixed BME Worst Health (*n* = 352) 9 Predictors (African-Caribbean, Bangladeshi, Pakistani and Other BME)	Direction of Relationship between Variables Better Health is Associated with:
**Individual Characteristics**	Physical Activity (1)	Physical Activity (1)	Physical activity (1)	Higher physical activity levels per week
	Age (2)	Age (7)	Age (2)	Younger in age
	-	Disability (6)	Disability (3)	Not having a disability
	-		Gender (8)	Being female
	Work Status (6)		Work Status (11)	Being in work
**Social Environment**	Trust (4)	Trust (8)	-	Greater levels of trust
	-	Companionship (2)		Greater companionship

	-	Feeling left out (4)	-	Not feeling left out
	-	Isolated (5)	-	Being less isolated
	-	Place Belonging (9)	Place Belonging (10)	Belonging to the neighbourhood
**Neighbourhood Environment**	Liveability (3)	Liveability (3)	-	Higher liveability of neighbourhood
	Satisfaction with area (7)	-	Satisfaction with area (4)	Higher satisfaction with neighbourhood
**Local Green Space (GS)**	-	-	GS Satisfaction (6)	Higher GS satisfaction
	-	-	GS Safety (7)	Higher GS safety
	Access to GS (5)	Access to GS (10)	-	Walking to GS
	-	-	Visit GS with someone (5)	Visiting GS with someone
	-	-	GS frequency visits (Winter) (9)	Visiting GS more frequently in winter
	-	Presence of another GS (11)	-	The presence of another local GS in neighbourhood

## Abbreviations

The following abbreviations are used in this manuscript:

AUC	Area under the curve
CCR	Correlated component regression
BME	Black and minority ethnic group
GS	green space
GH	general health
SE	standard error
Ns	non-significant

## Appendix A. Sampling Structure

The sampling structure was developed to reflect the strong historical, urban concentrations of particular BME groups, with a focus on the Midlands, Greater Manchester and London. Our sampling frame aimed for a minimum of two ethnic groups per case study location, with a minimum of 40 respondents per cell (12 no cells in all from 6 locations), with a target aim of 520 participants in total. Our target groups were: Bangladeshi, Pakistani, Indian, African-Caribbean, white British and other BME. The sampling specification for each case study area was achieved; a total of 523 participants completed the questionnaire with between 85 and 88 participants in each case study location (see Table A1 below). The ethnic groups were not evenly distributed across all cities but localized in particular cities and locations (wards) where the population of a target ethnic group was likely to be high and yield an adequate sample. For example, the Bangladeshi group all came from Greater Manchester (Oldham or Rochdale).

**Table A1:** Sample distribution by geographic location and ethnicity.

Location	Indian	Pakistani	Bangladeshi	African-Caribbean	White British	Other BME	Total
	Sample	Sample	Sample	Sample	Sample	Sample	
**London: Islington**				44		44	**88**
**London: Hackney**				17	44	27	**88**
**North West: Oldham**		43	45				**88**
**North West: Rochdale**		43	44				**87**
**Midlands: Wolverhampton**	28	14			35	8	**85**
**Midlands: Coventry**	29	15		2	35	6	**87**
**Total**	57	115	89	63	114	85	**523**

## Appendix B. CCR Regressions Predicting General Health for Each Health Segment

In the following sections, regressions are shown for general health data for each of the health groups. In each case the table gives the model fit for R squared and accuracy for both training and cross validation; followed by the standardized regression coefficients and their breakdown by components, and the Pratt Co-efficient.

**Table B1:** CCR model fit and standard coefficients for predictors of “very good” general health, people of Indian origin.

**Model Fit**	**Cross Validation**	**Standard Error**
R2	0.20	0.07
AUC	0.74	0.01
Accuracy	0.80	0.02
**Predictor Variables in Rank Order**	**Standard Co-Efficient**	**Contribution to Model (Pratt %)**
Physical activity	−4.52	26
Age	5.02	24
Liveability	3.69	19
Trust	0.40	2
Access to GS	0.29	1
Work status	3.31	10
Satisfaction with area	5.18	18

Note: The variables included in the model had a performance ranging from 1000 to 909 out of 1000 in out of sample regression runs.

**Table B2:** CCRs model fit and standard coefficients for predictors of “good” general health, white British people.

**Model Fit**	**Cross Validation**	**Standard Error**
R2	0.20	0.07
AUC	0.71	0.01
Accuracy	0.73	0.01
**Predictor Variables in Rank Order**	**Standard Co-Efficient**	**Contribution to Model (Pratt %)**
Physical activity	−0.56	18
Companionship	0.42	9
Liveability of Area	0.44	10
Feeling left out	0.40	9
Isolated	0.38	8
Disability	−0.38	8
Age	0.38	11
Trust	0.38	8
Place Belonging	0.35	7
Means to GS	0.34	6
Presence of another GS	0.34	6

Note: The variables included in the model had a performance ranging from 1000 to 955 out of 1000 in out of sample regression runs.

**Table B3:** CCR model fit and standard coefficients for predictors of “poor” general health, Mixed BME (people of African-Caribbean, Bangladeshi, Pakistani origin, Other BME).

**Model Fit**	**Cross Validation**	**Standard Error**
R2	0.23	0.07
AUC	0.81	0.01
Accuracy	0.76	0.01
**Predictor Variables in Rank Order**	**Standard Co-Efficient CC1**	**Contribution to Model (Pratt %)**
Physical activity	−1.98	26
Age	2.07	27
Disability	−1.43	10
Satisfaction with area	1.12	7
Visit GS with someone	−1.24	2
GS Satisfaction	−1.25	1
GS Safety	0.80	5
Gender	−1.03	6
GS frequency visits (Winter)	−0.86	7
Place Belonging	0.97	3
Work status	0.69	6

Note: The variables included in the model had a performance ranging from 1000 to 740 out of 1000 in out of sample regression runs.
